# Promoting physical therapists’ use of research evidence to inform clinical practice: part 3 – long term feasibility assessment of the PEAK program

**DOI:** 10.1186/s12909-016-0654-9

**Published:** 2016-05-12

**Authors:** Julie K. Tilson, Sharon Mickan, Robbin Howard, Jonathan C. Sum, Maria Zibell, Lyssa Cleary, Bella Mody, Lori A. Michener

**Affiliations:** Division of Biokinesiology and Physical Therapy, University of Southern California, 1540 Alcazar St., CHP 155, Los Angeles, CA 90089 USA; Gold Coast Health and Griffith University, Southport, 4215 QLD Australia; Agile Physical Therapy, 3825 El Camino Real, Palo Alto, CA 94306 USA

**Keywords:** Knowledge translation, Evidence based practice, Education, Post-graduate training, Physical therapy

## Abstract

**Background:**

Evidence is needed to develop effective educational programs for promoting evidence based practice (EBP) and knowledge translation (KT) in physical therapy. This study reports long-term outcomes from a feasibility assessment of an educational program designed to promote the integration of research evidence into physical therapist practice.

**Methods:**

Eighteen physical therapists participated in the 6-month Physical therapist-driven Education for Actionable Knowledge translation (PEAK) program. The participant-driven active learning program consisted of four consecutive, interdependent components: 1) acquiring managerial leadership support and electronic resources in three clinical practices, 2) a 2-day learner-centered EBP training workshop, 3) 5 months of guided small group work synthesizing research evidence into a locally relevant list of, actionable, evidence-based clinical behaviors for therapists treating persons with musculoskeletal lumbar conditions--the Best Practices List, and 4) review and revision of the Best Practices List, culminating in participant agreement to implement the behaviors in practice. Therapists’ EBP learning was assessed with standardized measures of EBP-related attitudes, self-efficacy, knowledge and skills, and self-reported behavior at baseline, immediately-post, and 6 months following conclusion of the program (long-term follow-up). Therapist adherence to the Best Practice List before and after the PEAK program was assessed through chart review.

**Results:**

Sixteen therapists completed the long-term follow-up assessment. EBP self-efficacy and self-reported behaviors increased from baseline to long-term follow-up (*p* < 0.001 and *p* = 0.002, respectively). EBP-related knowledge and skills showed a trend for improvement from baseline to long-term follow-up (*p* = 0.05) and a significant increase from immediate-post to long-term follow-up (*p* = 0.02). Positive attitudes at baseline were sustained throughout (*p* = 0.208). Eighty-nine charts were analyzed for therapist adherence to the Best Practices List. Six clinical behaviors had sufficient pre- and post-PEAK charts to justify analysis. Of those, one behavior showed a statistically significant increase in adherence, one had high pre- and post-PEAK adherence, and four were change resistant, starting with low adherence and showing no meaningful improvement.

**Conclusions:**

This study supports the feasibility of the PEAK program to produce long-term improvements in physical therapists’ EBP-related self-efficacy and self-reported behavior. EBP knowledge and skills showed improvement from post-intervention to long-term follow-up and a trend toward long-term improvements. However, chart review of therapists’ adherence to the participant generated Best Practices List in day-to-day patient care indicates a need for additional support to facilitate behavior change. Future versions of the PEAK program and comparable multi-faceted EBP and KT educational programs should provide ongoing monitoring, feedback, and problem-solving to successfully promote behavior change for knowledge translation.

## Background

Evidence based practice (EBP), the integration of research evidence, patient perspectives, and clinical expertise, has become a gold standard for physical therapist education and clinical practice around the world [1, 2]. Knowledge translation (KT) has gained international acceptance as a foundation for the successful integration of research evidence into complex healthcare environments [3]. Evidence of effective educational programs for promoting EBP and KT in physical therapy practice is limited [4, 5].

A recent systematic review by Dizon et al*.* found limited evidence to inform EBP education for allied health professionals [6]. While some interventions have shown short-term improvements in EBP knowledge, skills, and attitudes, there is a paucity of evidence regarding behavior change and long-term outcomes [6]. A systematic review by Menon et al. found limited evidence that active, multi-component KT interventions were effective for improving physical therapists’ knowledge of best practice and self-reported adherence to best practices for particular patient populations (e.g. persons at risk for falls, person with rheumatoid arthritis) [4]. Yet, no studies monitored actual adherence to best practices. Further, no studies have evaluated the impact of a combined EBP and KT intervention on EBP learning and adherence to best practices in patient care.

The Physical therapist-driven Education for Actionable Knowledge translation (PEAK) program is an educational program designed to promote physical therapists’ integration of research evidence into clinical decision-making [7]. A mixed methods analysis reported feasibility of the 6-month program based on therapist-participant focus groups and short term EBP learning outcomes [8]. However, further analysis is needed to understand the feasibility of this program for producing long-term benefits and improving therapist adherence to evidence based patient care.

Most assessments of EBP education, including our own previous analysis of the PEAK program, have focused on EBP-related attitudes, self-efficacy, knowledge, skills, and self-reported engagement in EBP behaviors [4, 6]; all related to the five steps of EBP [9] (i.e. asking clinical questions, searching for best available evidence, appraising research evidence, integrating evidence with clinical expertise and patient perspectives, and evaluating outcomes). The validity of assessing behavior solely through self-report of EBP implementation has clear limitations; observational measures of EBP behaviors are an important addition to assessment of behavioral change among clinicians [10, 11]. This study adds therapists’ adherence to a participant-generated list of best practices, to traditional measures of EBP learning to better understand the impact of the PEAK program on therapists’ success translating knowledge into day-to-day patient care.

A study to assess feasibility for implementing the PEAK program was conducted from 2010-2011 among physical therapists at the University of Southern California clinical practices. Previous reports describe the program and its theoretical underpinnings [7] and a mixed-method analysis of immediate post-PEAK outcomes [8]. The purposes of this manuscript are to report 1) long-term outcomes regarding therapists’ EBP-related attitudes, self-efficacy, knowledge and skills, and self-reported behaviors, and 2) therapists’ adherence to participant-generated, evidence-based behaviors in patient care.

## Methods

### Participants

Twenty-five physical therapists practicing in three geographically dispersed USC patient care centers (2 outpatient; 1 inpatient) were invited to participate through staff meetings and individual email. Therapists were required to have a minimum of 6 months clinical experience, be providing patient care at the University of Southern California physical therapy practices at least 20 h per week, be able to attend both days of an EBP knowledge and skills workshop, and be willing to commit to study activities at least 1 h per month for 6 months. The study was approved by the University of Southern California Health Science Campus Institutional Review Board (HS-10-00593). All participants consented to participate.

### PEAK program

**Fig. 1:**
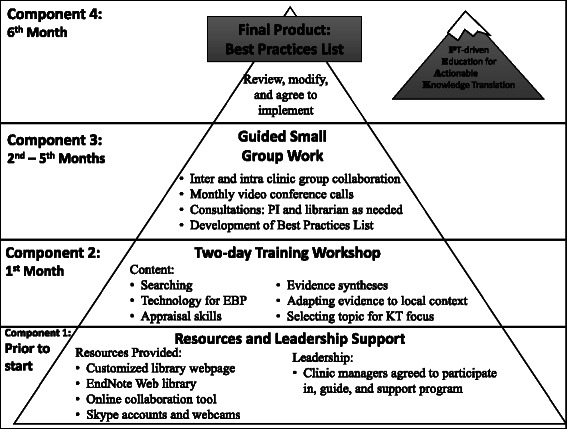
Timing and integration of components of the Physical Therapist-driven Education for Actionable Knowledge Translation (PEAK) program (figure reads from bottom to top). The program started with garnering support from clinic managers and placing links to technology resources at each facility’s computer work stations. Next, participants attended a two-day workshop addressing evidence based practice (EBP) and knowledge translation (KT) skills. Five months of guided small group work followed as participants developed the Best Practices List. In the final month, the Best Practices List was reviewed by expert faculty unaffiliated with the study. Finally, after multiple rounds of revisions, all participants agreed to implement the Best Practices List in their clinical practice. (Reproduced from: Tilson JK, Mickan S. Promoting physical therapists' of research evidence to inform clinical practice: part 1 – theoretical foundation, evidence, and description of the PEAK program. *BMC Med Educ.* 2014;14:125)

The PEAK program is a multifaceted, learner-centered education program designed to promote physical therapists’ use of research evidence in clinical decision-making. Its theoretical foundations, described previously [7], are in social cognitive [12, 13] and adult learning theories [14]. PEAK draws on two KT frameworks, Promoting Action on Research Implementation in Health Services [15] and the Knowledge to Action cycle [16] and is designed to help therapists overcome known barriers to EBP in physical therapy [17–21]. The program was 6 months in duration with four consecutive, interdependent components: 1) acquiring managerial leadership support and electronic resources in three clinical practices, 2) a two-day learner-centered EBP training workshop, 3) 5 months of guided small group work synthesizing research evidence into a locally relevant list of actionable, evidence-based clinical behaviors for therapists treating persons with musculoskeletal lumbar conditions--the Best Practices List, and 4) review and revision of the Best Practices List, culminating in participant agreement to implement the behaviors in practice. (Fig. 1, Table 1).

**Table 1:** Overview of the Physical therapist-driven Education for Actionable Knowledge translation (PEAK) learning objectives and four consecutive interdependent learning components

Learning Objectives
By the end of the intervention we expected that therapists would be able to:
1. Identify gaps in knowledge and develop focused, searchable clinical questions;
2. Find the best available evidence to inform their question using appropriate online databases;
3. Critically appraise the quality of found evidence;
4. Write succinct statements of locally recommended practices that integrate research evidence with their clinical expertise and knowledge of patient perspective; and
5. Integrate newly learned skills and behaviors into their everyday work habits.
Additionally, from an organizational perspective, we expected that, at the conclusion of the PEAK program, all therapists would:
1. Agree to follow the common set of locally generated, evidence-based, best practices that they had developed, for a specific group-selected patient population;
2. Engage in activities to support using research to inform clinical practice for other patients; and
3. Demonstrate implementation of research within their clinical practice.
Instructor
The instructor for the program was the study principal investigator (PI; JKT) – a physical therapist with 10 years experience teaching EBP and promoting KT in clinical and classroom environments.
Component 1
Prior to starting the PEAK program leadership support was secured by engaging managers of the three, geographically separate physical therapy service departments (2 outpatient, 1 inpatient) at the University of Southern California (USC) to contribute to logistical organization of the PEAK program and to participate throughout the program. Resources for supporting the integration of research in practice were provided to all participants as follows:
• A custom library web page developed and maintained by a medical librarian to reflect key online resources
• A group online reference manager account (EndNote Web® [Thompson Reuters])
• An online collaboration tool (Backpack™, 37 Signals, LLC) was purchased and set-up for all participants to use (a research assistant managed organization of the collaboration tool)
• Skype™ (Microsoft Skype Division) accounts were established for each facility, including purchase and installation of webcams to facilitate inter-facility web conferencing
Links to online resources were installed as bookmarks on each participant’s work computer.
Component 2
During the first month of the program participants attended a two-day workshop that combined didactic and active learning around topics of EBP and KT including:
• Review of the 5-step EBP model (1 h)
• Searching skills (3 h; PubMed, National Guidelines Clearinghouse, Translating Research Into Practice, PEDro)
• Appraisal skills (3 h; primary studies of interventions, systematic reviews, and clinical practice guidelines)
• Integrating research evidence with patient perspectives and clinical expertise (1 h)
• Using technology to keep up to date (2 h: podcasts, myNCBI auto-searches, RSS feeds, etc.; study-specific tools: Backpack™, EndNote Web®, Skype™)
• Selection of clinical area and five sub-topics around which a list of locally relevant evidence-based best practices would be generated (2 h)
• Initiation of small group work for developing the Best Practices List (2 h)
A librarian attended one day of the workshop to promote participants’ use of library resources and was available for consultation throughout the course of the educational program. A copy of the educational materials used for the 2-day workshop is available from the corresponding author.
It is important to note that the *participants* selected the clinical area that would be pursued for the rest of the program based on their common interests and a perceived opportunity for patient benefit. Further, participants identified five sub-topics of the clinical area and organized themselves into five corresponding small groups based on the sub-topic(s) of greatest interest to each participant.
Component 3
For 5 months following the workshop, participants met regularly in small groups (three to seven therapists) to develop a list of locally relevant ‘best practices’ for their clinical sub-topic. A designated group leader accepted responsibility for organizing regular small group communication and monthly reporting to the larger group. Each small group worked through the five EBP steps to find, appraise, and synthesize the highest quality research evidence for their clinical sub-topic. More specifically, groups were tasked to use research evidence, their own expertise, and knowledge of patient perspectives to generate actionable, evidence-based behaviors that could be implemented in their own practice. Actionable, evidence-based behaviors submitted by each small group were compiled into a single, “Best Practices List” for all participants to implement.
Small groups determined how often they met (virtually or in person) and used the online collaboration tool to accomplish their work. Monthly lunchtime meetings were conducted using Skype™ video conference for all participants to report on and discuss their progress. Monthly meetings were facilitated by the study principal investigator (PI) and attended by the study librarian. The study principal investigator and librarian met individually with groups when requested.
Component 4
At the end of the 5^th^ month, each small group submitted between 7 and 15, actionable, evidence-based behaviors to the Best Practices List. The study PI compiled the behaviors and distributed them to all participants for review and comment. Two rounds of review and comment were conducted online. Next, the list was sent for external review by experts selected by participants. Expert feedback was incorporated into the Best Practices List and at the end of the 6^th^ month, participants attended a final two hour meeting to review and discuss each behavior. Edits were made until all participants were satisfied that they could adhere to the recommended practice. At the conclusion of this final meeting the study participants gave verbal affirmation that they agreed with and would follow the behaviors outlined in the Best Practices List. This final list (online Appendix) was published in booklet form and distributed electronically and in hard copy to all participants.

Extracted with permission from: Tilson JK, Mickan S. Promoting physical therapists' of research evidence to inform clinical practice: part 1 – theoretical foundation, evidence, and description of the PEAK program. *BMC Med Educ.* 2014;14:125

All components of the PEAK program support a participant-driven learning experience – working as a group to generate a Best Practice List around a common, participant-selected clinical area. The Best Practices List is a locally generated list of evidence-based, actionable behaviors that participants agree (as a group) to implement in their clinical practice. Participants self-organized into small groups to review literature around their chosen topic of musculoskeletal lumbar conditions and generate evidence-based actionable behaviors. The actionable behaviors were reviewed and revised through a process of peer and expert review until all participants felt that they could implement the Best Practices List in practice [8]. The final Best Practices List, published previously as electronic supplementary material [8], consisted of 38 total behaviors divided among five sub-topics: outcome measures (three behaviors), non-specific low back pain (ten behaviors), post-surgical lumbar conditions (five behaviors), stenosis (nine behaviors), and spine tumors (11 behaviors). Each participant verbally agreed to implement all behaviors in their clinical practice [7, 8]. The final list was distributed to each participant electronically and to each site in bound hard copy.

### Evaluation of the PEAK program

**Fig. 2:**
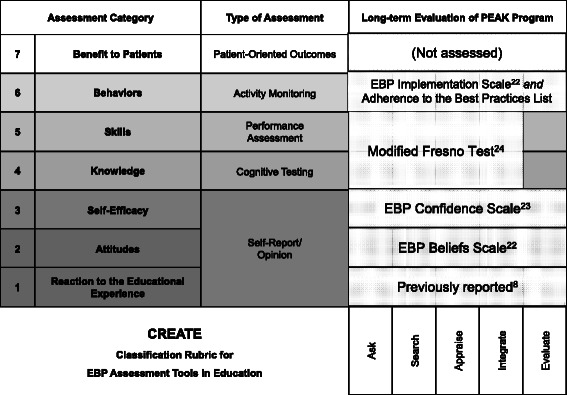
Standardized measures of EBP learning used to assess long-term feasibility of the PEAK program overlaid on the Classification Rubric for EBP Assessment tools in Education. Two standardized assessments were modified: 13 EBP-specific items were used from the EBP Implementation Scale and six attitude-specific items were used from the EBP Beliefs Scale. Figure modified with author permission from Tilson J, Kaplan S, Harris J, et al. Sicily statement on classification and development of evidence-based practice learning assessment tools. *BMC Med Educ.* 2011;11(1):78

The Classification Rubric for EBP Assessment Tools in Education (CREATE) model [11] provided the theoretical framework for evaluating the feasibility of the PEAK program. CREATE was designed to guide comprehensive and systematic evaluation of complex EBP-related educational programs like PEAK. It identifies seven categories for evaluating EBP and KT educational curricula (Fig. 2). We used four standardized measures of EBP learning to assess therapists’ EBP-related attitudes, self-efficacy, knowledge and skills (combined), and self-reported behavior. We used therapist adherence to the Best Practices List behaviors, assessed through chart review, to determine whether the program resulted in changes in therapists’ clinical behavior with patients.

#### EBP learning: standardized measures

**Fig. 3:**
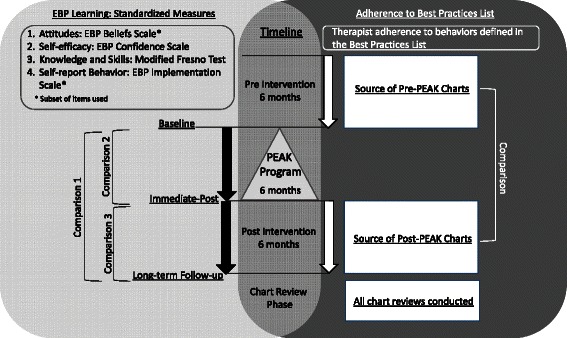
Study timeline including administration of standardized measures of EBP learning and indication of time periods for which charts were reviewed for therapist adherence to the Best Practices List

Standardized measures of EBP learning were assessed at baseline, immediately-post PEAK program, and 6 months later for long-term follow-up (Fig. 3).

*Attitudes* toward EBP were assessed using the attitudes items from the EBP Beliefs Scale. The 16-item EBP Beliefs Scale measures EBP attitudes and self-efficacy and has demonstrated construct and criterion validity among practicing nurses [22]. To exclusively measure attitudes about EBP, we summed responses to six Likert-type items from the EBP Beliefs Scale (1, 4, 5, 9, 11, and 13); higher scores (total possible = 30) indicate more positive attitudes.

*Self-efficacy* for EBP was assessed using the Evidence-based Practice Confidence Scale. This measure has established face and content validity among healthcare professionals, including physical therapists [23]. The Evidence-based Practice Confidence Scale consists of 11 items with responses ranging from 0 to 100 % confidence (in 10 percentile increments); responses are averaged to generate a mean confidence between 0 and 100 %.

*Knowledge and skills* for EBP were assessed with the 13-item modified Fresno Test which has demonstrated reliability and content and construct validity among physical therapists [24]. The modified Fresno Test consists of open-ended questions scored with a standardized rubric and results in scores from 0 to 224; higher scores represent better knowledge and skills.

Self-reported EBP *behavior* was assessed using the EBP Implementation Scale which has demonstrated construct and criterion validity among nurses [22]. The 18-item EBP Implementation Scale assesses implementation of EBP and the collection, analysis, presentation, and reaction to patient data. Because the PEAK program did not ask participants to collect, analyze, present, or react to patient data, 5 items addressing these behaviors (5, 7, 15–17) were not relevant to our question and risked masking any observable changes in self-reported EBP behavior. Therefore, to exclusively measure self-reported behaviors associated with the 5-steps of EBP, we summed responses to 13 items from the EBP Implementation Scale (1–4,6,8–14,18); higher scores (total possible = 65) indicate greater frequency of EBP implementation.

#### Adherence to the best practices list

Therapist adherence to the Best Practice List was assessed via medical chart review of therapists’ documented practice behaviors in the 6 months prior to and following the PEAK program (Fig. 3). Raters were two doctor of physical therapy (DPT) students who participated in 6 h of training and demonstrated 96 % and 95 % accuracy for extracting data from 10 randomly selected charts compared to the primary investigator. Charts meeting all of the following criteria were eligible for review:

1. Diagnosis: The patient’s primary diagnosis for the episode of care was related to a musculoskeletal lumbar spine condition. Charts with the following ICD-9 codes as a primary diagnosis were reviewed for inclusion: 721.0–9 spondylosis and allied disorders; 722.0–9 intervertebral disc disorders; 724.0–9 other and unspecified disorders of back.
2. Therapist: The therapist responsible for the majority (>50 %) of the patient’s care participated in the PEAK program.
3. Date: Therapy ended in the 6 months prior to therapists participating in the PEAK program (pre-PEAK charts). Therapy began in the 6 months following the PEAK program (post-PEAK charts).

For the two outpatient facilities, potential charts were identified for review through a single electronic database. Chart review was then conducted with paper charts. For the inpatient facility, potential charts were identified through a second database. The inpatient charts had been converted to electronic archives, but did not contain therapy evaluation and care notes. Without basic therapy information electonically, hard copy charts from among approximately 5000 patients needed to be individually retrieved from an archive center. Given that the return on this search was expected to be about 20 charts (0.4 %), it was not feasible to review the inpatient paper charts. Thus, inpatient charts were not included in the analysis.

Raters manually reviewed eligible charts for therapist adherence to behaviors listed in the Best Practices List. Behaviors from the Best Practices List sub-topic ‘outcome measurement’ were relevant to all charts. Behaviors from the remaining four sub-topics were only relevant when the appropriate diagnosis (non-specific low back pain, post-surgical lumbar conditions, stenosis, and lumbar spine tumor) was present. Adherence was recorded as either ‘behavior present’, ‘behavior not present’, or ‘not applicable’. ‘Not applicable’ was used when a therapist would not be expected to adhere to a behavior because the patient case did not fit pre-defined criteria for that behavior (e.g. behaviors relevant only to patients with *acute* non-specific low back pain were recorded as ‘not applicable’ in the instance of a patient with *chronic* non-specific low back pain).

### Analysis

The analysis consisted of two parts: standardized measures of EBP learning and therapist adherence to the Best Practices List. Only therapist participants that completed baseline, immediate-post, and long-term assessments were in included in either analysis.

The first analysis assessed therapist outcomes on four standardized measures of EBP learning at baseline, immediately-post PEAK program, and long-term follow-up. Descriptive statistics were used to describe therapist participants. One-way repeated measures ANOVA was used to identify change in each standardized measure. Pair wise comparisons for statistically significant repeated measures ANOVA were assessed with Bonferroni correction. A *post hoc* one-way repeated measures ANOVA of the standardized measures, restricted to those therapists whose charts were included in the chart review portion of the study, was also conducted.

The second analysis assessed therapist adherence to the Best Practices List pre- and post-PEAK. Descriptive statistics were used to report the proportion of charts from pre- and post-PEAK timeframes in addition to the proportion of charts associated with each sub-topic of the Best Practices List and each outpatient therapist. Mean age, number of physical therapy visits, and number of comorbidities was assessed for the patients whose charts were included in the analysis. Chi-square test was used to identify differences in adherence to the Best Practices List between pre- and post-PEAK charts. Based on a sample size analysis with 80 % power to detect a 50 % change in adherence at alpha = 0.05, statistical analyses were conducted only for behaviors with ≥14 applicable charts in each of the pre and post groups. Bonferroni correction was used to account for multiple comparisons within the chart review analysis.

## Results

### Participants

Eighteen physical therapists met the inclusion criteria and agreed to participate. Two left USC-employment prior to the long-term follow-up leaving 16 therapists with a complete data set for analysis (Table 2).

**Table 2:** Participant characteristics

Variable^a^	Count or mean (sd)
N	16
Age, mean (range)	35.2 (27–51)
Years in Practice, mean (range)	8.1 (2–20)
Professional Designation	
Staff Physical Therapist	12 (75.0 %)
Clinic Manager	4 (25.0 %)
Highest Degree	
Doctor of Physical Therapy	13 (81.3 %)
Masters	3 (18.7 %)
Clinical Hours per week	
11–20 h^b^	2 (12.5 %)
21–30 h	2 (12.5 %)
31–40 hours	7 (43.4 %)
>40 h	5 (31.3 %)
Primary Clinic Setting	
Outpatient	9 (56.3 %)
Inpatient Acute	7 (43.8 %)

^a^Variables are reported as count and percentage unless otherwise noted

^b^Two therapists initially met the inclusion criteria of >20 clinical hours per week but experienced a decrease in clinical hours during the course of the study due to changes in responsibilities

### EBP learning: standardized measures

**Fig. 4:**
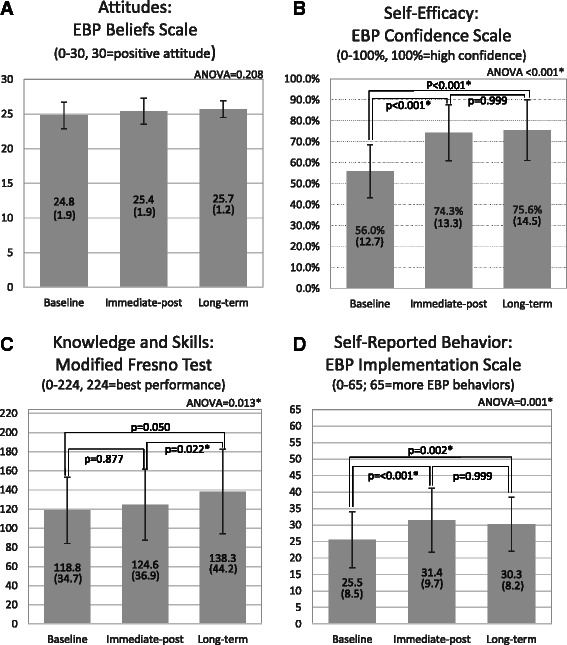
Mean scores at baseline, immediate-post, and long-term follow-up (6 months after conclusion of PEAK program) for each standardized measure of EBP learning; standard deviation in parentheses. Results of one-way ANOVA are listed in the top right for each assessment. Paired comparisons are illustrated by brackets over bars. ‘*’ Indicates statistical significance in ANOVA or pair-wise comparisons with Bonferroni correction. (Note: Two standardized assessments were modified: 13 EBP-specific items were used from the EBP Implementation Scale and six attitude-specific items were used from the EBP Beliefs Scale)

Results of the four standardized measures of EBP learning for baseline, immediate-post, and long-term follow-up are detailed in Fig. 4. Attitude scores started high and were still high at long-term follow-up (Fig. 4, panel a, *p* = 0.208).

Improvement from baseline to long-term follow-up was observed for EBP self-efficacy (Fig. 4, panel b, *p* < 0.001) and self-reported EBP behaviors (Fig. 4, panel d, *p* = 0.002). Change for these two measures occurred between baseline and the immediate-post follow-up. No significant gain or loss was observed from immediate-post follow-up to long-term follow-up. EBP knowledge and skills showed a trend toward improvement from baseline to long-term follow-up (Fig. 4, panel c, *p* = 0.05). While change was not significant from baseline to immediate-post follow-up (*p* = 0.877), a significant improvement was noted from immediate-post to long-term follow-up (*p* = 0.022).

### Chart reviews

**Fig. 5:**
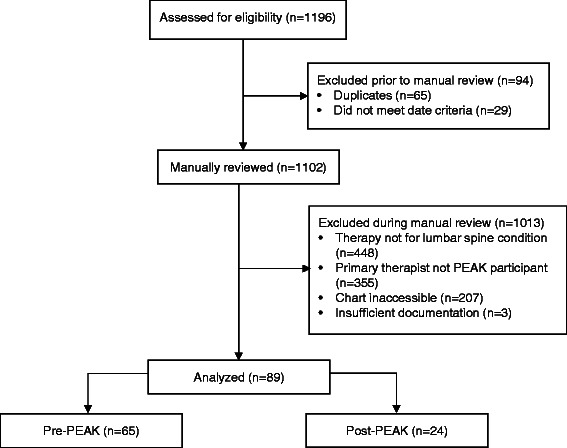
Flow chart of charts eligible for review to assess therapist adherence to the Best Practices List pre- and post-PEAK

One thousand one hundred ninety six potential charts were screened for inclusion (Fig. 5). Of those, 89 (7.4 %) met the inclusion criteria and were reviewed for adherence to the Best Practices List behaviors (Table 3). Charts from the pre-PEAK time period made up 73 % (*n* = 65) of all eligible charts. Most charts reviewed were associated with patients with non-specific low back pain (78 charts, 86.6 %).

**Table 3:** Chart review (*n* = 89 charts)

Variable	Number of charts (%)^a^
N	89
Time	
Pre-PEAK	65 (73.0 %)
Post-PEAK	24 (27.0 %)
Sub-topic	
Outcome measures	89 (100 %)
Non-specific low back pain	78 (87.6 %)
Post-surgical lumbar conditions	7 (7.9 %)
Stenosis	4 (4.5 %)
Lumbar spine tumor	0
Outpatient therapist^b^	
Therapist 1	15 (16.9 %)
Therapist 2	4 (4.5 %)
Therapist 3	11 (12.4 %)
Therapist 4	12 (13.5 %)
Therapist 5	6 (6.7 %)
Therapist 6	12 (13.5 %)
Therapist 7	10 (11.2 %)
Therapist 8	19 (21.3 %)
Characteristics of patients whose charts were included in the analysis	
Age	Mean (sd) or Range
Mean (sd)	50.0 (19.2)
Range	20–87
Number of comorbidities	
Mean (sd)	2.7 (2.4)
Range	0–13
Physical therapy visits	
Mean (sd)	11.0 (8.7)
Range	1–48

^a^Variables are reported as count and percentage unless otherwise noted; PEAK = Physical therapist-driven Education for Actionable Knowledge translation, sd = standard deviation

^b^One therapist who participated in PEAK and was seeing patients full time during the chart review data collection period was not the primary therapist on any charts that met the inclusion criteria

#### Behaviors

Six of the 38 behaviors from the Best Practices List met the sample size criteria (≥14 charts in both the pre- and post-PEAK time period) and were assessed for change in therapist adherence (Table 4). Of those six, one behavior, ‘depression screen’, had a statistically significant change; adherence increased from 55.4 to 95.8 % (*p* < 0.001). The behavior involving ‘provision of a progressive home exercise program for persons with chronic LBP’ had high adherence pre-PEAK (92.7 %) that was sustained post-PEAK (87.5 %). The remaining four behaviors had <50 % adherence pre-PEAK that did not improve post-PEAK.

**Table 4:** Adherence to select behaviors from the Best Practices List (6 of 38 behaviors with ≥14 applicable charts pre- and post-PEAK)

Best Practices List sub-topic	Behavior	Adherence (count/applicable charts^a^)		*p*-value
		Pre	Post	
Outcome Measurement	The modified Oswestry Disability Index should be administered at the beginning and end of treatment [33, 36, 37].	7.7 % (5/65)	12.5 % (3/24)	0.482
	Fear Avoidance Beliefs Questionnaire administered at the beginning and end of treatment [37].	6.2 % (4/65)	4.2 % (1/24)	0.718
	A depression screen should be conducted with the following two questions (1) “During the past month, have you often been bothered by feeling down, depressed, or hopeless?” and (2) “During the past month, have you often been bothered by little interest or pleasure in doing things?” [38].	55.4 % (36/65)	95.8 % (23/24)	<0.001
Non-specific Low Back Pain	Patients with non-specific LBP should be assessed for lumbar instability based on the following criteria: (1) positive prone instability test; (2) positive (>6/9) Beighton scale; (3) aberrant movement patterns (instability catch, Gower sign); (4) production of pain with mobilization of hypermobile segment (especially L4-5, and L5-L1); and/or (5) presence of excessive lumbar mobility (excessive lumbar flexion/reversal of lumbar lordosis) [37, 39, 40].	44.1 % (26/59)	33.3 % (6/18)	0.151
	For patients with chronic (>12 weeks) LBP, a progressive exercise program (neuromuscular control, strength, and endurance) should be provided. If a patient meets this criterion but is not provided with progressive exercises, the reason should be documented [41].	92.7 % (38/41)	87.5 % (14/16)	0.563
	For patients with chronic (>12 weeks duration) non-specific LBP, a multidisciplinary rehabilitation program (including exercise, psychological pain management, back school, PT/OT, psychology/psychiatry, and medical management) should be considered. Therapists should document discussion of the appropriateness of such an intensive program with patients with chronic non-specific LBP [42].	9.8 % (4/41)	12.5 % (2/16)	0.297

^a^The number of applicable charts varies depending on patient criteria (e.g. diagnosis, acuity) specified in some Best Practice List behaviors

#### *Post Hoc* analysis

An analysis of standardized measure performance restricted to the therapists whose charts contributed to the chart review data (*n* = 8) was consistent with results observed in the full cohort of 16 therapists.

## Discussion

The PEAK program resulted in long-term improvements in EBP-related self-efficacy and self-reported behaviors, which were evident immediately post intervention and maintained for 6 months without further intervention. EBP knowledge and skills showed improvement from immediate-post to long-term follow-up. Therapists’ attitudes about EBP started positive and stayed positive throughout the study. However, analysis of medical records suggests that the PEAK program did not provide sufficient support to facilitate therapists’ improved adherence to the Best Practices List. Of the six behaviors analyzed from the Best Practices List, one showed a statistically significant increase in adherence, one had high pre- and post-PEAK adherence, and four were change resistant, starting with low adherence and showing no meaningful improvement.

### EBP learning: standardized measures

The long-term improvement in EBP self-efficacy and self-reported behavior supports feasibility of using the PEAK program for improving EBP and KT among physical therapists. These improvements were first noted directly after the PEAK program and were maintained independently by therapists for 6 months beyond the completion of the program. This pattern can be explained using social cognitive theory which posits that increased self-efficacy for a given task will result in increased time and energy that an individual will devote to that task. For example, Salbach and colleagues’ found that physical therapists with higher self-efficacy for EBP are more likely to engage in EBP behaviors [17, 25]. Few other studies of EBP and KT programs have assessed self-efficacy as a separate domain from attitudes and beliefs about EBP as a method of practice [4, 5, 26]. However, an international consensus statement suggests that self-efficacy and attitudes represent separate learning domains [11] and a validated self-efficacy assessment tool is available [23]. We propose that future studies assess self-efficacy distinctly from other elements of ‘attitude’ to determine how changes in self-efficacy and self-reported behavior might be linked.

We also found improvements in EBP knowledge and skills; there was a trend toward improvement from baseline to long-term follow-up and a significant change from immediate-post to long-term follow-up. It is important to note that this change occurred at a time when there was no active intervention, suggesting that clinicians’ increased self-efficacy and behavioral changes may have led to improved knowledge of EBP. It is worth noting that the absolute baseline to long-term improvement (19.5 points) fell short of the 25.7 point minimal detectable change derived for the modified Fresno Test [27]. However, our therapists’ mean baseline score (118.8) was consistent with third-year doctor of physical therapy students (118.5) while their mean long-term follow-up score (138.3) approached that of EBP-expert faculty (149.0), suggesting that meaningful change may have occurred [24]. Dizon et al. [6] found an increase in physical therapists’ knowledge and skills based on the adapted Fresno Test [28] (similar to the modified Fresno Test but excludes statistical questions) immediately after a one-day training workshop. However, in contrast to our program, that increase degraded over the following 3 months despite ongoing educational support. Adult learning theory [14] suggests that increased experience leads to greater resources for learning. The difference between our results and Dizon’s findings may be due to the prolonged and self-directed nature of the PEAK program. Our findings suggest that as therapists progressively increased their confidence and use of EBP over the course of the year, they may have accumulated improvements in EBP knowledge and skills that were detectable between the immediate-post and long-term follow-up. We recommend including long-term follow-up for analysis of similar programs.

Our finding of consistently positive attitudes about EBP reflects a common finding among physical therapists [19, 21, 29]. Thus, addressing attitudes around EBP may not be a critical component of educational programs for physical therapists.

### Adherence to best practices list

To our knowledge this is the first study to objectively assess physical therapists’ patient care behavior in response to a prolonged, multi-faceted program promoting general EBP and KT as opposed to a KT intervention specific to implementing a particular externally-generated clinical practice guideline [4, 5]. Two studies found improved physical therapist adherence to specific clinical practice guidelines after a KT intervention [30, 31]. In contrast, we saw improvement in adherence, as measured by medical chart review, to only one of the Best Practices List behaviors among the six with sufficient charts for analysis.

Though it stands alone among the six behaviors analyzed, the substantial improvement observed in therapists’ adherence to conducting a depression screen can be explained by the influences of the PEAK program. The depression screen questions were part of an intake form that patients filled out before their first physical therapy visit. The intake form and associated procedures did not change during the study time period. However, our chart review showed that patients were inclined to leave the questions blank. It may be that as a result of developing the Best Practices List, therapists were more likely to follow-up with patients who left the questions blank thus resulting in a substantial increase in adherence to collecting this information.

Four of the six Best Practices List behaviors analyzed were change resistant, therapists had low pre- and post-PEAK adherence. The “behavior change wheel” developed by Michie and colleagues [32] provides insight into how the PEAK program may have fallen short in helping therapists translate these evidence based behaviors into practice. The behavior change wheel identifies three elements--capability, motivation, and opportunity--as the central ingredients needed for behavior change. The PEAK program offered support for *capability* to change by increasing therapists’ EBP-related knowledge and skills for conducting the best practices. Further, our previous analysis [8] suggests that PEAK promoted *motivation* by creating a group culture energized around the idea of improving patient care through EBP. However, the PEAK program likely fell short in addressing *opportunity*.

Opportunity refers to those factors that lie outside of the individual. While organizational support was carefully cultivated for the overall PEAK program, no specific organizational support was garnered to facilitate and sustain implementation of the Best Practices List. For example, therapists agreed to administer the modified Oswestry Disability Index [33] at the beginning and end of treatment. However, no logistical issues were addressed regarding administrating of the questionnaire: Who would provide patients with the questionnaire? How would the questionnaire be collected if a patient unexpectedly stopped therapy? How would an alert be generated if a questionnaire was missing? The Knowledge to Action Cycle identifies that organizational support including monitoring, feedback, and problem solving with therapists is needed to sustain knowledge implementation [16]. The additional provision of organizational support after the identification of the Best Practices may have resulted in improved therapist adherence to the change resistant behaviors.

It is important to note that sustained low adherence to four of the six Best Practices List behaviors does not inherently mean that therapists somehow misrepresented their self-reported EBP behaviors. Based on our previous analysis, therapists reported increased behaviors associated with asking clinical questions, searching for and reading research evidence, and discussing their findings with patients and peers [8]. This is consistent with activity diaries that showed increased EBP behaviors (formulating PICO questions, searching, appraising, and applying) after a similar EBP educational program [6]. These behaviors are important precursors to actually changing what is done with patients. Additionally, medical record data can over or under-represent what is actually done in practice [34]. It is possible that therapist behavior changed more than their documentation revealed. Ultimately, our data seems to indicate that more was needed to convert EBP behaviors into observable changes in patient care.

### Limitations

This study concludes evaluation of the PEAK program, a theoretically driven, evidence-based educational intervention to promote EBP and KT among physical therapists practicing in a geographically dispersed academic medical center. The feasibility nature of the study called for a small sample size of therapist participants in the educational program. This fact combined with a first attempt to monitor therapists’ adherence to the participant-generated Best Practices List revealed a number of challenges. Lack of efficiently searchable electronic records made the hospital charts with therapists’ adherence data inaccessible. Additionally, although lumbar spine conditions in general represent about 25 % of ambulatory visits to physical therapists [35], our participants saw a relatively small number of people with three of the Best Practice List sub-topics (stenosis, post-surgical lumbar conditions, tumor-related lumbar conditions). Additionally, the number of charts that met our inclusion criteria post-PEAK was substantially smaller than the pre-PEAK cohort. This appears to have been due to chance as work flow in the outpatient clinics was not noticeably different during those two time periods. Although the chart review sample size was small, we feel that the absolute rates of adherence support the statistical findings and that the chance that a change in adherence occurred and was missed (type II error) is low. It is important to recognize, however, that further study with larger groups of therapists from diverse settings and a larger number of analyzed charts are needed to support generalizability of our findings. It is also important to consider that we did not assess patient outcomes. We are unable to say if patients experienced benefit from therapists’ participation in the PEAK program.

Previously identified limitations [8] that warrant consideration include the fact that the academic environment provided therapist participants with medical library resources not standard in many general practice settings. Further, therapists practicing in an academic setting are potentially more likely to embrace efforts to promote EBP and KT. Finally, because single domain outcome measures for EBP attitudes and behaviors (specific to the 5-steps of EBP) were not available, we modified the EBP Beliefs Scale and EBP Implementation Scale, by excluding certain items, to meet our needs. The resulting scales had strong face validity but further validation is needed, particularly as evaluation of the PEAK program moves beyond the feasibility stage.

## Conclusion

This study supports the feasibility of the PEAK program to produce long-term improvements in physical therapists’ EBP-related self-efficacy and self-reported behavior. EBP knowledge and skills showed improvement from post-intervention to long-term follow-up and a trend toward long-term improvements. However, chart review of therapists’ adherence to the participant-generated Best Practices List in day-to-day patient care indicated a need for additional support to facilitate behavior change. Future versions of the PEAK program and comparable multi-faceted EBP and KT educational programs should include monitoring, feedback, and problem solving, to promote therapists’ adherence to best practices in patient care.

### Ethics approval and consent to participate

The study was approved by the University of Southern California Health Science Campus Institutional Review Board (HS-10-00593). All participants consented to participate.

### Consent for publication

Not applicable.

### Availability of data and materials

All data is available through the first author of the manuscript.

## Abbreviations

CREATE: classification rubric for EBP assessment tools in education
EBP: evidence based practice
KT: knowledge translation
PEAK: physical therapist-driven education for actionable knowledge translation
